# Development of tolerance to chemokine receptor antagonists: current paradigms and the need for further investigation

**DOI:** 10.3389/fimmu.2023.1184014

**Published:** 2023-07-28

**Authors:** Patrick Grudzien, Henry Neufeld, Mbasogo Ebe Eyenga, Vadim Gaponenko

**Affiliations:** Department of Biochemistry and Molecular Genetics, College of Medicine, University of Illinois at Chicago, Chicago, IL, United States

**Keywords:** chemokine receptors, antagonist, antagonist tolerance, clustering, oligomerization, signaling

## Abstract

Chemokine G-protein coupled receptors are validated drug targets for many diseases, including cancer, neurological, and inflammatory disorders. Despite much time and effort spent on therapeutic development, very few chemokine receptor antagonists are approved for clinical use. Among potential reasons for the slow progress in developing chemokine receptor inhibitors, antagonist tolerance, a progressive reduction in drug efficacy after repeated administration, is likely to play a key role. The mechanisms leading to antagonist tolerance remain poorly understood. In many cases, antagonist tolerance is accompanied by increased receptor concentration on the cell surface after prolonged exposure to chemokine receptor antagonists. This points to a possible role of altered receptor internalization and presentation on the cell surface, as has been shown for agonist (primarily opioid) tolerance. In addition, examples of antagonist tolerance in the context of other G-protein coupled receptors suggest the involvement of noncanonical signal transduction in opposing the effects of the antagonists. In this review, we summarize the available progress and challenges in therapeutic development of chemokine receptor antagonists, describe the available knowledge about antagonist tolerance, and propose new avenues for future investigation of this important phenomenon. Furthermore, we highlight the modern methodologies that have the potential to reveal novel mechanisms leading to antagonist tolerance and to propel the field forward by advancing the development of potent “tolerance-free” antagonists of chemokine receptors.

## Chemokine receptors: functions and pharmacology

Chemokine receptors belong to a subfamily of rhodopsin-like class A G-protein coupled receptors. Their primary function is to orchestrate directional migration of cells (chemotaxis) in response to stimulation by small extracellular proteins (chemokines). Chemokines are secreted at the sites of infection or inflammation and create a concentration gradient that is sensed by the chemokine receptors on the surface of immune cells. Activation of chemokine receptors promotes engagement of immune cells in the inflammatory response. Recognition of chemokine receptors by chemokines is frequently promiscuous with several different chemokines being able to bind and activate the same receptor. However, some chemokine receptors have monogamous cognate ligand binding partners rather than being promiscuous.

While obtaining insight into activation of chemokine receptors from X-ray structures has been challenging due to difficulties associated with crystallization of chemokine-bound receptors, mechanistic details are emerging with the advent of cryo-electron microscopy and advanced crystallography studies. The structures of agonist-occupied CCR2, CCR5, viral chemokine receptor US28, and CXCR2 (1–5) are consistent with the previously proposed two-step, two-site mechanism (6, 7). This mechanism involves the initial recognition of the chemokine by the flexible N-terminus of the receptor. The encounter complex matures after the chemokine inserts its N-terminal region into the helical bundle of the receptor, shifting its conformational ensemble towards the active state characterized by repositioning of helix 6. While the general features of activation are preserved among chemokine receptors, for which structural information is available, the interactions of chemokines and their receptors differ in detail. For example, the N-terminus of CXCL8 does not penetrate deep into the helical bundle of CXCR2, but, instead, interacts with a shallow pocket, primarily establishing contacts with electrostatic residues in TMs 5 and 6 (5). Differences in chemokine:receptor interaction modes suggest the existence of several distinct activation mechanisms. Conventional chemokine receptors in the active state accelerate the exchange of GDP for GTP in the coupled Gα (primarily Pertussis toxin sensitive Gαi), while the atypical chemokine receptors ACKRs do not. Instead, the ACKRs tend to act as chemokine scavengers but can also modulate the function of conventional chemokine receptors through heterodimerization (8). Interestingly, the structures of agonist-bound ACKR3 resemble those of active conventional chemokine receptors (9). However, distinct conformations and dynamics of ACKR3’s intracellular loops and the more compact cytoplasmic pocket may explain its bias against G-protein activation.

**Figure 1:**
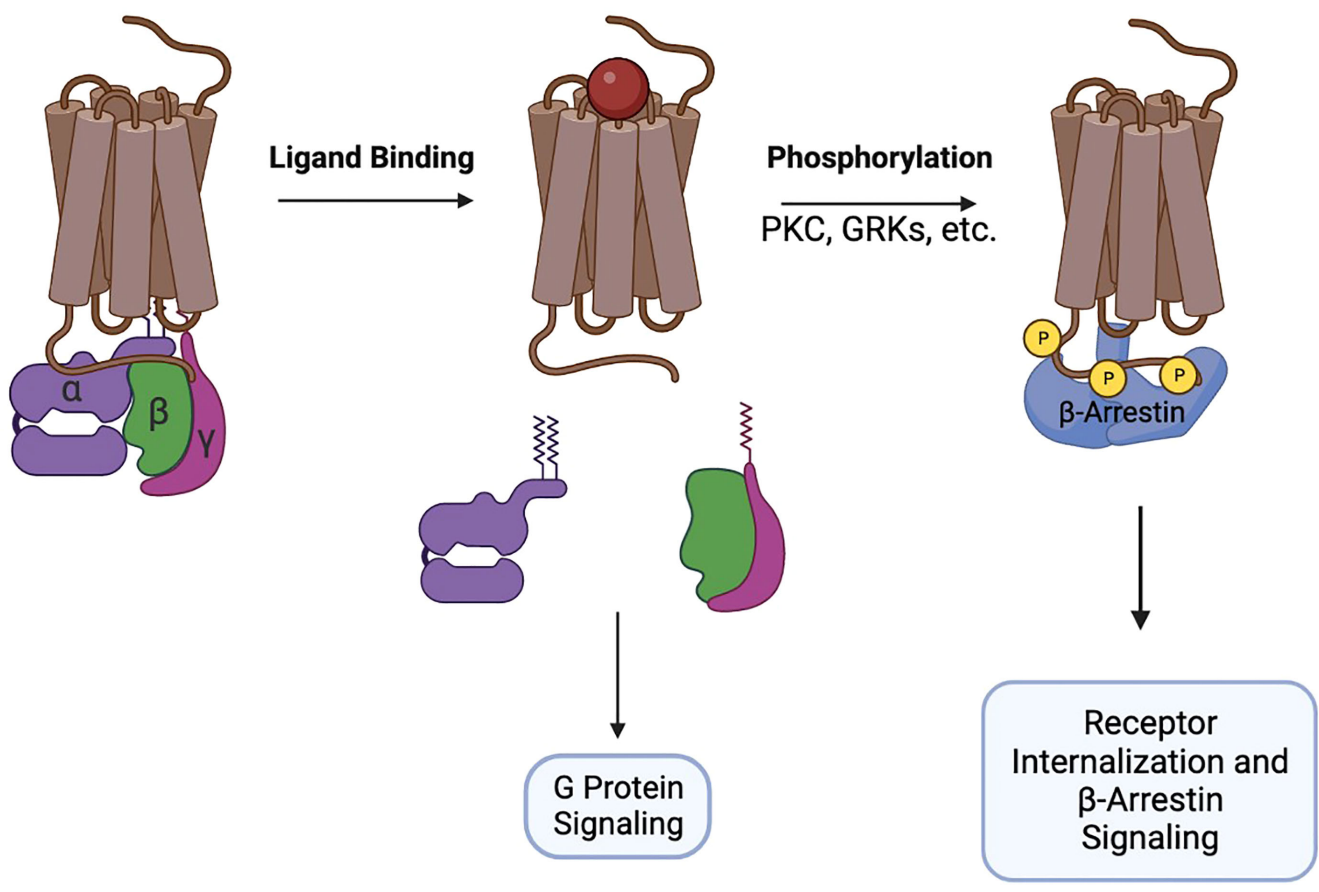
Brief schematic of GPCR pharmacology.

GTP-loaded Gαi disengages from the receptor and from the Gβ/γ heterodimer and interacts with adenylyl cyclase to inhibit production of cAMP. At the same time, Gβ/γ activates MAPK, PI3K, phospholipase C, Ca2+ flux, and promotes remodeling of the cytoskeleton necessary for the formation of cellular protrusions. Together, the signaling of Gα and Gβ/γ initiates chemotaxis of cells towards increased concentrations of the chemokines. After G-protein signaling is initiated, chemokine receptors recruit GRK, PKC, and Pim1 kinases to their intracellular portions and undergo phosphorylation. The phosphorylation sites attract β-arrestins 1/2 that interact with membrane phosphoinositides and assemble the clathrin machinery, promoting receptor endocytosis and desensitization (10, 11). Additionally, β-arrestins participate in signal transduction, primarily through the MAPK pathway (12) (**Figure 1**).

The concept of homologous (limited to the stimulated receptor) (13–15) and heterologous (includes the stimulated receptor and other receptors) (16, 17) desensitization is well studied. Chemokine receptor desensitization is the result of prolonged contacts with the agonist, leading to reduced cell surface receptor levels and activation. Mechanistically, desensitization is linked to rapid receptor internalization followed by degradation or by slow repopulation of the cell surface by the recycled or newly synthesized receptors. This process can be influenced by the extracellular environment, receptor type, and the exact effectors and downstream signaling involved in receptor function (13). Desensitization was found to be ligand type dependent for chemokine receptors that have multiple ligands. One of the best examples of this phenomenon is CCR4 that can be activated by either CCL22 or CCL17 (18, 19). CCL22 stimulates CCR4 desensitization at a much higher level compared to CCL17 (20). This sensitivity to chemokine types might be required for the sequential functions of CCL17 and CCL22, allowing for precise recruitment of Th2 lymphocytes into tissues (20).. Heterologous desensitization of chemokine receptors can be exemplified by the cross-talk between opioid and chemokine receptors (21). For instance, pretreatment of cells with RANTES, a chemokine for CCR5 or with CXCL12, a CXCR4 specific chemokine, reduces the subsequent efficacy of DAMGO, µ-opioid receptor’s agonist. Remarkably, simultaneous treatment with chemokine and µ-opioid receptor agonists produced a significantly greater effect, indicating a rapid desensitization of the opioid receptor. This reaction also works in the opposite direction, where the activation of the µ-opioid receptor desensitizes the chemokine receptors, but to a lesser degree (22).

Selective activation of either G-proteins or receptor internalization can be achieved through biased agonism, a phenomenon that has received significant attention in the context of chemokine receptors (23–25). Biased agonists have been identified for multiple chemokine receptors, such as CXCR4 (26), CXCR3 (27, 28), CCR2 and CCR5 (29). There are additionally a few instances of a natural ligand or receptor modifications that stimulate biased signaling. Specific examples can be found in the N-terminal modifications of CCL15, a CCR1 ligand (30), and TM helical modifications of CCR5 (31). Structural characterization of two CCR1-bound truncation variants of CCL15 (one balanced agonist and one biased agonist) revealed differences in their binding modes and resulting receptor conformations (32). The residue Y291 in helix 7 of CCR1 acts as a “toggle switch” for biased signaling. Binding of the balanced agonist causes Y291 to form hydrogen bonds with Y113 in helix 3 and Y255 in helix 6, promoting the conformation of the receptor favorable for recruitment of β-arrestin. However, upon binding of the biased agonist, Y291 exists in two alternative conformations, one resembling the balanced agonist bound state and one resembling the apo-state of the receptor. Thus, CCR1 bound to the biased agonist displays reduced recruitment of β-arrestin (32).

Partial agonists that induce incomplete response in the stimulated receptor have been described for multiple chemokine receptors (29, 33). In some cases, these are promiscuous chemokines that induce more robust responses in the context of other receptors (34, 35) or chemokines with altered N-termini (36–40). The presence of subdued functional outcomes in chemokine receptors stimulated with partial agonist chemokines suggests several possible receptor binding modes that engage alternative mechanisms of activation. Sometimes, the partial agonistic activity of chemokines is related to their ability to form homo-dimers (41–43), while small molecule and peptide partial agonists likely employ allosteric mechanisms (44, 45).

Inverse agonists of chemokine receptors that inhibit their basal activity by stabilizing the inactive conformations can be successful therapeutics. For example, maraviroc, an inverse agonist of the HIV cellular entry portal CCR5, is approved by the FDA for treatment of AIDS (46, 47). Motixafortide, an inverse agonist of CXCR4, has been successfully tested in clinical trials and might gain FDA approval as a hematopoietic stem cell mobilizing agent (48). Other inverse agonists of chemokine receptors have been identified and some of these might also lead to the development of promising therapeutics (49) or used as tools to study the functions of chemokine receptors (50–52). Inverse agonists of chemokine receptors utilize allosteric mechanisms of action (53–55). The mechanistic structural data describing the inhibitory activity of maraviroc has been particularly insightful (3, 56, 57). Maraviroc’s phenyl group interacts with hydrophobic residues in helices 3 and 6 of CCR5 and prevents the downward movement of helix 6 that is necessary for receptor activation (57). The phenyl group also blocks the activation-promoting signal transmitted through M287 in helix 7 (56, 58).

The ability of chemokine receptors to stimulate chemotaxis of cells explains their role in multiple diseases (59). In acute and chronic inflammatory disorders, chemokine receptors drive excessive chemotaxis of leukocytes to the sites of inflammation. These disorders include asthma, acute respiratory distress syndrome, autoimmune diseases, such as multiple sclerosis and rheumatoid arthritis, and others (60–65). The critical importance of proper management of these conditions is highlighted by the devastating COVID-19 pandemic where the most common comorbidities were linked to the proinflammatory state (66). Chemokine receptors participate in the pathogenesis of cardiovascular disease by recruiting leukocytes to the areas of arterial damage and by promoting smooth muscle migration into the intima and thrombus formation over atherosclerotic plaques (67). Chemokine receptors play an important role in viral and bacterial infections. Chemokine receptors facilitate HIV cellular entry (68), and mediate exacerbation of immune response in coronavirus (69) and Ebola infections (70). Normal chemokine receptor signaling is subverted by poxviruses and herpesviruses that induce production of viral chemokines and chemokine receptors (71). Chemokine receptors balance pathogenic and protective immune responses in Mycobacterium tuberculosis infections (72). Chemokine receptors are also key mediators of cancer-related inflammation and can promote angiogenesis, tumor growth, and metastasis (73). Thus, it is not surprising that chemokine receptors are important therapeutic targets in many diseases. However, the development of chemokine receptor antagonists has been challenging primarily due to toxicity and low efficacy of these molecules in clinical trials (74). While toxicity of chemokine receptor antagonists is likely related to their promiscuous binding to secondary targets and associated off-target effects, the issue of low efficacy is harder to explain. One potential reason for the low efficacy in clinical trials is divergent functions of chemokine receptors in animals used for the preclinical development and in humans. In addition, we previously observed that after prolonged treatment with antagonists of chemokine receptors CXCR4 and CCR3, cells eventually became tolerant to the antagonists and were able to mount a robust chemotactic response even in the presence of the inhibitors (75, 76). Antagonist tolerance can also explain multiple failures in clinical trials with chemokine receptor antagonists, particularly those that reported low efficacy of the molecules under investigation. Although antagonist tolerance has been reported for several GPCRs (77–80), this phenomenon remains understudied and the underlying mechanisms are not delineated. Here, we summarize the available knowledge on antagonist tolerance in the context of therapeutic targeting of chemokine receptors and encourage deeper investigation of this problem.

Optimism in the community remains high as there are multiple groups currently studying chemokine receptors. Seven clinical trials testing potential therapeutics targeting chemokine receptors are ongoing at the time of this publication, and there have been over 30 since 2005, twelve of which specifically focused on chemokine receptor antagonists. Despite much effort and significant financial investment, only three drugs have ever been approved for clinical use. These three (plerixafor, maraviroc and mogamulizumab) have proven therapeutic efficacy for their approved purpose despite the development of tolerance.

Plerixafor, a CXCR4 antagonist, releases hematopoietic progenitors from the bone marrow by blocking binding of the chemokine CXCL12 to the receptor and inhibiting downstream signaling. Mobilizing these cells allows their collection and purification from peripheral blood for transplantation. Plerixafor, also known as AMD3100 or Mozobil, is additionally used to treat non-Hodgkin’s lymphoma or multiple myeloma (81). Plerixafor is the only clinically approved CXCR4 antagonist, which inhibits downstream activation of G-proteins and β-arrestin. Apheresis occurs 11 hours after administration of the drug. Plerixafor displaces stroma-attached progenitor cells by blocking CXCL12 from homing cells to the bone marrow. This prevents progenitor cells from returning to the bone marrow, where they develop chemoresistance. The short treatment time for Plerixafor may aid in overcoming tolerance related issues, however, in cases of longer treatment times tolerance development remains a concern. Drugs similar to AMD3100, like AMD11070 and Filgrastim, which suffer from prolonged apheresis fall victim to tolerance (82, 83). Mogamulizumab is another anti-chemokine/anti-CCR4 antibody that targets lymphomas as well, and has been clinically approved for Cutaneous T-cell lymphomas and Adult T-cell leukemia/lymphoma (84). This drug increases antibody-dependent cellular cytotoxicity (ADCC) (73). Mogamulizumab increases ADCC by high-affinity binding with the Fc receptor on effector cells (85). Mogamulizumab inherently deals with the common problem of tolerance by labeling the tumor cells expressing CCR4 for destruction through the mechanism of ADCC.

The third and final chemokine receptor drug approved by the FDA through clinical trials is maraviroc. This drug targets the cellular entry of HIV by interfering with the coreceptor CCR5 (86–88). Other indicators of success are a high nadir CD4 cell count, detectable viral load, protease inhibitor exposures and young age. Maraviroc is always used in combination with other HIV drugs and, therefore, has its own challenges in determining efficacy and tolerance. The combination of drugs may create unintended or exacerbate existing issues potentially speeding up tolerance development on top of other negative effects.

Addressing chemokine receptor tolerance should be essential when considering efficacy of antagonists but is often overlooked. Low efficacy is a surprisingly frequent problem during chemokine antagonist clinical trials because the preclinical phase of development is expected to be highly selective for efficacious drugs. This being said, there are a number of possible reasons for the low efficacy, tolerance being one of these. Tolerance, manifested by cell surface receptor accumulation is cited as a reason for the low efficacy of AMD3100 in prolonged treatments (75), but this idea of tolerance is hardly mentioned elsewhere. In many completed and terminated chemokine antagonist clinical trials, efficacy is investigated but not the specific factors leading to inefficacy are not mentioned or remain overlooked. For example, the CXCR2 antagonist AZD5069 was studied in its role in controlling severe exacerbations in patients with asthma in a clinical trial by AstraZeneca (89). It was determined that CXCR2 antagonist did not reduce the frequency of severe exacerbations, but no connection was made to specific mechanisms. Another case of inefficacy is demonstrated by chemokine CCR2 receptor antagonists. Although the preclinical phase of development should select highly efficacious and selective drugs, efficacy became a major issue in NCT00992186, which studied a biased antagonist (90). PF-04634817, designed to treat Diabetic Macular Edema was said to be well tolerated with a high level of CCR2 antagonism, inefficacy was a major issue, with no reasons concluded. Carlumab, another CCR2 antagonist also had problems with inefficacy, with 0 complete or partial responses in reducing tumors. The list goes on with similar outcomes, inefficacy was observed but no conclusion was made regarding the contributing factors leading to this result.

Investigation into lack of efficacy is oftentimes overlooked leading to a loss of usable information for future studies where the same factors, tolerance included, could be having a significant impact. Another issue plaguing clinical trials is that efficacy and tolerance are often disguised by other problems, such as species-dependent biology or disease pathogenesis, resulting in unsuccessful clinical trials. In a clinical trial, NCT01160224, for an oral CCR3 antagonist, GW76694, 68% of subjects reported adverse events. The issue of efficacy is grouped into broader categories of why the drug is ineffective without further exploration into the mechanistic effects of the antagonist itself. These broader categories, involving questions regarding the role of CCR3 in the pathogenesis of asthma, potentially overshadow the molecular mechanisms at play, such as tolerance. This is just another example of a lack of inquiry into the biochemical basis for the failure of clinical drugs. To more coherently investigate chemokine antagonists, reasons for inefficacy must be thoroughly investigated. Through investigations such as those done by Hitchinson *et* al (75) tolerance will likely have an important impact. By considering lack of efficacy as contingent of tolerance, the current paradigms of chemokine receptor antagonism may be challenged. The mechanisms at play by antagonists within GPCR pharmacology is much less understood compared to the abundant agonist information. This is likely a major obstacle to the successful development of chemokine receptor antagonists.

## Antagonist tolerance

Although antagonist tolerance in the context of chemokine receptors is not well documented, there are multiple reported cases regarding the development of tolerance to antagonists of other GPCRs. A large portion of these studies focuses on tolerance to antipsychotics, specifically those targeting Dopamine receptors. These studies tend to only examine clinical occurrence and symptoms as a result of tolerance but do not focus on the mechanisms of antagonist tolerance (91–93). The few studies beyond the scope of antipsychotics explore tolerance as a secondary aim, usually in cases where the use of antagonists is preceded by inverse agonist treatments.

An important GPCR target of investigation of antagonist tolerance has been the Histamine H_1,2,3_ receptor. Cases exploring histamine receptor antagonists acting as inverse agonists oftentimes connect this activity to the development of tolerance (94–96). This view considers tolerance as part of a larger mechanistic process where the mode of the interaction of pharmacological agents with their targets depends on the cellular environment, previous treatments, and changes over time. These molecules tend to be evaluated for potential use against various diseases (97–99) without addressing the reasons for failure to produce new and effective antagonists that can succeed in clinical trials. Even among studies exploring the difficulties in developing antagonists and ways to overcome these challenges (100, 101), investigation of antagonist tolerance is often omitted. A good launch point is applying FDA-approved antagonists of chemokine receptors to situations that require prolonged use and asking questions about tolerance and its mechanisms (75). However, many more studies need to be done. Given the lack of information specifically regarding tolerance to antagonists of chemokine receptors, in this review we will explore how cellular responses to antagonists of other GPCRS may be mechanistically informative or related to the development of tolerance to antagonists of chemokine receptors.

## Mechanistic insights into antagonist tolerance

### Similarities between antagonist tolerance and agonist tolerance

Given significant gaps in knowledge of antagonist tolerance, it is helpful to compare it to a better studied phenomenon of agonist tolerance. There is an excellent in-depth review by Raehal et al. (102) that discusses the development of agonist tolerance to opioids. The primary concepts that are relevant to both agonist and antagonist tolerance are regulation of receptor levels on the cell surface and complexity of the signaling cascades related to both events. Raehal et al. discuss how different drugs for the same opioid receptor lead varying levels of tolerance development. Typically, agonist tolerance is associated with a reduction in receptor levels on the cell surface, while in antagonist tolerance receptor levels on the cell surface rise. The conformational state induced by the agonist, signaling pathway stimulated and secondary or off-target effects all play key roles in determining the timeline and the severity of tolerance. As a brief example; DAMGO, a drug that induces a significant phosphorylation of the µ-opioid receptor utilizing GRK2 has been shown to produce lessened tolerance as compared to morphine, which has been shown to promote phosphorylation of the µ-opioid receptor through GRK and PKC (103). The idea that signaling, cellular environment and conformational state influence the presence and severity of tolerance is one we believe applies to antagonist tolerance as well.

### Timeline for tolerance

The time needed for the development of antagonist tolerance is likely dependent on many factors, including the antagonist type, the dosing regimen, and the lack for target selectivity. For example, the tolerance to Biperiden, a selective antagonist of the muscarinic M1 receptor, begins to develop clinically around the 3^rd^ night of drug administration (104). Clozapine, an antipsychotic, which targets the dopamine D2 receptor begins to exhibit reduced effectiveness within 2 days and its potency gradually decreases further (105, 106). However, other antipsychotics targeting the same receptor, such as Thioridazine, show a slight reduction in efficacy beginning at 4 days with a very slow development of tolerance afterwards (106). Two of the most frequently prescribed antipsychotics, Haloperidol and Risperidone, are heralded for their ability to avoid tolerance in patients. However, the progressive nature of the side-effects, associated with reduced on-target efficacy, suggests the potential involvement of antagonist tolerance (107–109). Tolerance development for both typical and atypical antipsychotics has been partially associated with the dosing method (intermittent vs continuous) as well as the non-specific binding events with the serotonin 5-HT receptor, ideas that we will discuss further in this review (108, 110). The phenomenon of tolerance is a crucial variable when considering the treatment of psychosis and other mental disorders and this concept should be applied to other GPCRs, especially chemokine receptors. When it comes to chemokine receptor antagonist tolerance, CXCR4-expressing Jurkat cells develop tolerance to Plerixafor (AMD3100) after 72 hours of treatment (75).

### Cell surface density increases in antagonist tolerance

In all cases of reported tolerance to antagonists targeting GPCRs, including chemokine receptors, an increase in receptor density on the cell surface is invariably observed. Although, there may be several different mechanisms leading to increased receptor expression in antagonist tolerance, it is likely related to inhibition of receptor turnover by antagonists that can inhibit recruitment of β-arrestin to the receptor. Internalization is a key regulatory mechanism for transmembrane receptors (111). Endocytosis maintains an appropriate concentration of receptor at the cell surface to prevent overpopulation, which can impact receptor function and signaling (112). GPCRs and especially chemokine receptors are fairly prone to hetero- and homo-oligomerization at high receptor density; both processes can significantly affect receptor signaling and regulation (113, 114). It is not surprising that directly affecting the cell surface concentration and receptor trafficking mechanisms may have effects on receptor oligomerization and structure. A prime example of antagonist-induced receptor density increases comes from observations by O’Dowd et al. who explored Dopamine D1, D5, and 2 serotonin receptors antagonist-induced cell surface density changes (115). They found that antagonist’s dose-dependently increases receptor density up to 15-fold. Even at lower antagonist concentrations, up to 8.5-fold density increases were seen. Hess et al. observed the effects of a dopamine D1 and D2 antagonist, cis-flupentixol on receptor upregulation and cataleptic effects of the drug (116). Researchers found that there was a significant upregulation of D2 receptors following treatment with cis-flupentixol. Moreover, the time to cataleptic effects in rats was increased, leading to the conclusion that upregulation of the receptor was associated with antagonist tolerance. Hitchinson et al. came to a very similar conclusion, suggesting that prolonged treatment of cells with Plerixafor (AMD3100) increased CXCR4 on the cell surface and led to the development of antagonist tolerance. Thus, there is significant evidence linking tolerance to increased levels of receptors on the cell surface after prolonged administration of the antagonists.

### Potential oligomerization of receptors in antagonist tolerance

The mechanistic importance of GPCR oligomerization has been a long-studied topic and yet its functional significance is not firmly established. Recent studies have suggested that oligomerization of GPCRs plays key roles in signaling and regulation (117, 118) and this sentiment has been echoed as especially important for chemokine receptors (114, 119). It is also emphasized that the formation of oligomers may impact drug discovery, as targeting a receptor dimer or even trimer may require adaptations for ligands, which only target monomeric receptors (120, 121). As we described previously, changes in receptor cell surface density have been observed after treatment with antagonists and this has been correlated to tolerance. This increase in receptor density likely elevates receptor oligomerization, which may be the underlying cause of the observed antagonist tolerance. This receptor oligomerization can be in the form of homo- or hetero-oligomers, both with a significant potential to alter receptor structure and function. Examining available structures of oligomerized CXCR4 reveals structural changes caused by oligomerization (122). These changes in structure can remodel the drug binding sites and reduce drug binding, leading to tolerance. In addition to forming homo-oligomers, evidence suggests GPCRs can readily hetero-oligomerize at the cell surface, such as the dopamine D_2_/Adenosine A_2a_, CXCR4/ACKR3 (atypical chemokine receptor 3), Serotonin 5_HT_/mGlu2, and many others. At high receptor cell surface density, hetero-oligomers can co-exist with homo-oligomers, further altering receptor signaling and responses to treatment with antagonists. To explore the potential effects of this increased heteromerization we will take a look at two systems, the Adenosine A_2a_ and Dopamine receptor interactions and CXCR4 interactions with multiple other GPCRs.

In the case of heteromeric A_2a_/D_2_, the A_2a_ receptors directly interact with and antagonize dopamine D_2_ receptors (123–125). Further exploration has found that targeting of A_2a_ with agonists can decrease D_2_ signaling and treatments with adenosine antagonists can increase D_2_ signaling, such interactions can also be seen in A_1_/D_1_ heteromers (123). This interaction not only impacts the signaling and function of the complexed receptors but also extends to ligand binding. There have been a few unique changes in ligand binding events observed from the interaction between A_2a_ and D_2_, namely, an allosteric network, which had 2 distinct effects: changed A_2a_ antagonists to have agonist activity and the appearance of modulated D_2_ agonist and antagonist affinity and efficacy (125).

Similarly, the concept of heteromerization can be applied in the context of a chemokine receptor and antagonist pair. CXCR4 has many heteromerization partners, including but not limited to the atypical chemokine receptor ACKR3, alpha- and beta-adrenergic receptors and CXCR3 (126–128). Early studies of Plerixafor asserted that it was selective to CXCR4 (129, 130), however, other studies found that it also acts as an ACKR3 agonist (131). Given that Plerixafor can target ACKR3 and CXCR4, which can form a heteromeric complex, there is a possibility for additional ligand effects and signal dampening. ACKR3 can also act as an allosteric modulator of CXCR4 (126). ACKR3 alters CXCR4 signaling in cancer by changing ligand binding, internalization, and signal propagation (132, 133). Remarkably, co-transfection of CXCR4 and ACKR3 caused degradation of CXCR4 and internalization of ACKR3 (134) and increased front cell velocity (135). Hetero-oligomerization of chemokine receptors, much like homo-oligomerization, can have significant impacts on the ligand binding and signaling pathways. When considering drug discovery, any changes to the receptor density and availability of heteromeric partners can influence the efficacy of drugs and lead to the development of unintended consequences, such as tolerance.

## Addressing antagonist tolerance

Chemokine receptor antagonists tend to be unable to maintain their therapeutic efficacy when prolonged (three days or longer) administration is necessary. Equipotent inhibition of chemotaxis and receptor endocytosis by unbiased antagonists may be the root for this progressive reduction in efficacy over time. Unlike unbiased antagonists, biased antagonists selectively inhibit G-protein signaling, while leaving β-arrestin recruitment and receptor internalization intact. Thus, biased antagonists do not lead to receptor accumulation on the cell surface over time and can avoid the development of tolerance. For example, the peptide R321 targeting the eosinophil chemokine receptor CCR3 acts as a biased antagonist by blocking G-protein signaling and eosinophil recruitment into the lungs. This biased antagonist then still allows β-arrestin recruitment and receptor endocytosis (76). Currently, there are no clinically approved CCR3 antagonists: antagonist tolerance may have been the cause of failure of the previously tested CCR3 antagonist in clinical trials against asthma. However, biased antagonists of CCR3 may be a promising alternative. Like R321, the CXCR4 biased antagonist X4-2-6 permits β-arrestin recruitment and receptor internalization but inhibits the G protein signaling. The G-protein signaling is blocked by the simultaneous interaction of X4-2-6 with CXCR4 and CXCL12, leading to a partial expulsion of CXCL12’s N-terminus from the helical bundle of the receptor. The remaining contacts between CXCL12 and the receptor allow activation of β-arrestin recruitment. The biased antagonists have the potential to address antagonist tolerance in the context of chemokine receptors by preventing signaling but permitting endocytosis, which prevents receptor accumulation on the cell surface. This mechanism of tolerance avoidance by X4-2-6 and R321 was tested by comparing potency between the novel peptides and unbiased antagonists before and after prolonged treatments. For example, the ability of X4-2-6 and Plerixafor to inhibit CXCR4-mediated chemotaxis of Jurkat T lymphocytic leukemia cells before and after the 72-hour treatment was determined. While the 72 hour exposure to Plerixafor led to a significant increase in the drug’s IC50 value for the inhibition of CXCL12-induced chemotaxis (75), cells treated with the biased antagonist X4- 2-6, had similar IC50 with and without pretreatment with the peptide. A very similar outcome was obtained with the CCR3 biased antagonist R321 in AML14.3D10-CCR3 cells. Both R321 and X4-2-6 are derived from the second transmembrane helix of their respective chemokine receptors and also contain sequences derived from the extracellular loop 1 of CXCR4 and CCR3 (75, 76). The loop sequences likely facilitate binding of the chemokines, while the sequences corresponding to the transmembrane regions can interact with the receptors. Chemokine binding even in the absence of the receptors might contribute to the inhibitory activity of the peptides through the potential chemokine sequestration mechanism, complicating delineation of contribution of biased antagonism to successful blocking of chemokine receptor signaling. Thus, further studies are needed with simpler peptides and/or small molecule biased antagonists to understand how the biased mechanism of chemokine receptor inhibition prevents antagonist tolerance.

Although biased antagonism is an effective approach to avoid tolerance, there may be additional ways to accomplish similar outcomes. Homo- and hetero- oligomerization of chemokine receptors might be directly related to the development of antagonist tolerance. A potential methodology to overcome tolerance would be inhibiting the formation of receptor oligomers. Drug discovery related to disruption of oligomers is already underway and has produced reliable data suggesting the potential druggability of oligomerization interfaces (136, 137). The logical idea of disrupting oligomer formation is utilizing peptides synthesized from the transmembrane helices of GPCRs. A review by Gallo et al (138) does an excellent job in discussing the current progress in peptide development and how researchers are slowly addressing the pitfalls of using peptides as drugs. The review discusses multiple successful attempts at using TM synthetic peptides to disrupt the formation of oligomers, such as A_2A_R homodimers and the cannabinoid receptor 2/5-hydroxytryptamine:2A heterodimer (139). The use of TM synthetic peptides as drugs is still a growing idea but the preliminary data gives an optimistic outlook on the future.

## Concluding remarks

**Figure 2:**
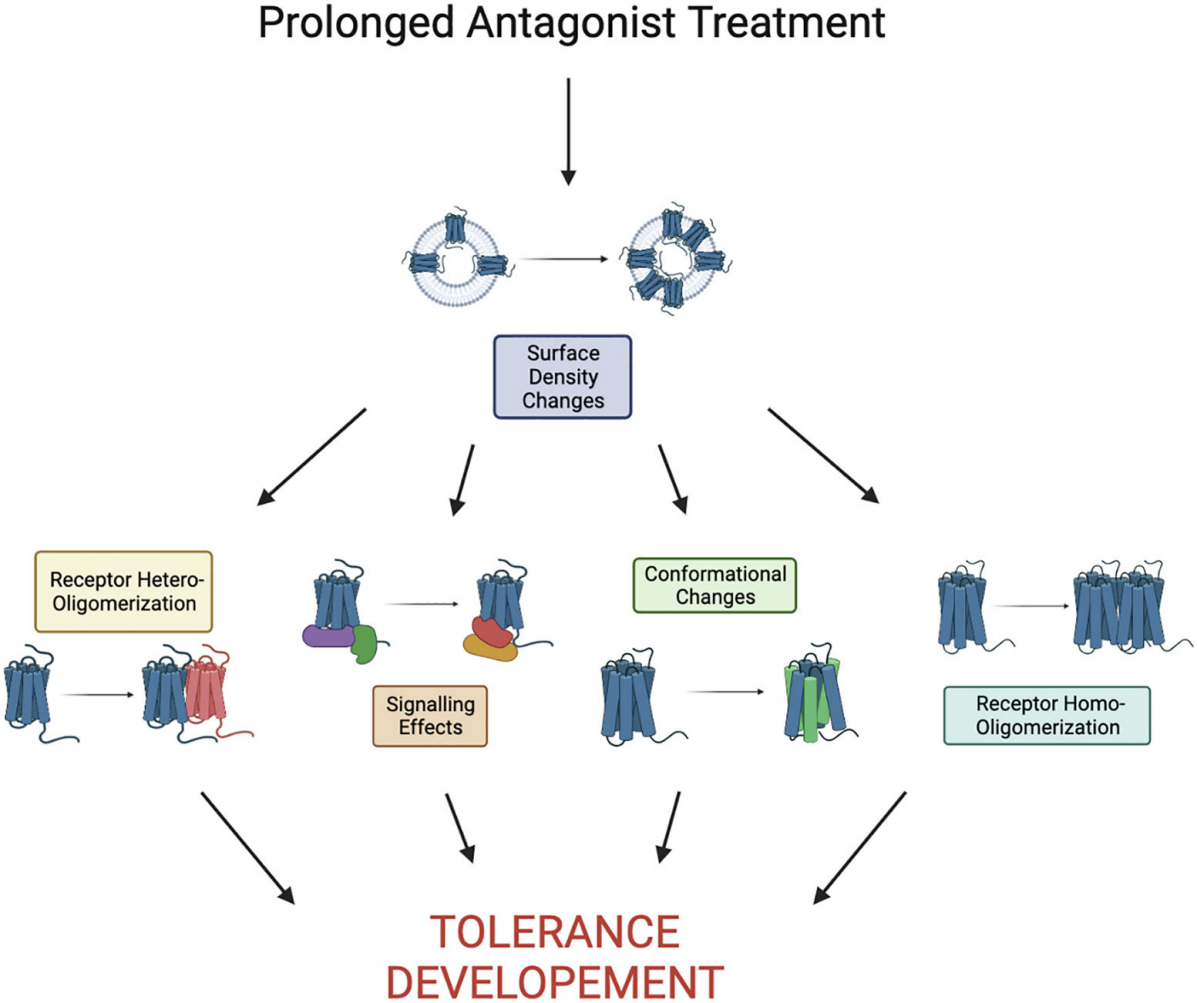
Proposed pathway to tolerance as a result of prolonged antagonist treatment. Here we define prolonged as the time it takes to see development of tolerance, it is largely receptor/drug dependent. Development of tolerance may be a result of any combination or all of the shown effects.

Tolerance to chemokine receptor antagonists remains unaddressed and this likely impedes successful development of chemokine receptor inhibitors for clinical use. To date, there is no comprehensive mechanistic understanding of antagonist tolerance. However, some clues can be derived from a handful of studies on chemokine receptors and other GPCRs (**Figure 2**). Antagonist tolerance tends to be accompanied by receptor accumulation on the cell surface possibly due to inhibition of receptor turnover by the antagonists. Thus, we propose that high receptor density plays a key role in the mechanism of tolerance. This is different from the low receptor levels at the cell surface commonly observed in agonist (primarily opioid) tolerance, which has different mechanisms. It seems likely that accumulation of chemokine receptors in the plasma membrane leads to their clustering and oligomerization. Under these conditions, rising numbers of receptor oligomers can far exceed the numbers of receptor oligomeric assemblies commonly seen in untreated cells and start to alter signaling pathways in response to drugs. As only three chemokine receptor antagonists have been approved for clinical use, different approaches to inhibition of chemokine signaling are sorely needed. Biased antagonists that block G-protein signaling but allow receptor internalization have been reported to avoid antagonist tolerance. There are likely other approaches to either avoid or alleviate tolerance. It will be interesting to test if inhibitors of receptor oligomerization or blockers of signaling associated with tolerance can be successful alternatives to conventional chemokine receptor antagonists. Given the wide array of diseases and conditions associated with chemokine receptors, more study into the mechanisms of tolerance will propel the development of therapeutics targeting chemokine receptors.

## Author contributions

VG conceived the idea for the article, wrote the introduction, and edited the manuscript. PG wrote 75% of the manuscript, generated a summary figure, and included references. HN wrote 25% of the manuscript. ME provided clinically relevant comments on the manuscript. All authors contributed to the article and approved the submitted version.

## References

[1] Shao Z Tan Y Shen Q Hou L Yao B Qin J. Molecular insights into ligand recognition and activation of chemokine receptors CCR2 and CCR3. Cell Discovery (2022) 8(1):44. doi: 10.1038/s41421-022-00403-4 35570218 PMC9108096

[2] Isaikina P Tsai CJ Dietz N Pamula F Grahl A Goldie KN. Structural basis of the activation of the CC chemokine receptor 5 by a chemokine agonist. Sci Adv (2021) 7(25). doi: 10.1126/sciadv.abg8685 PMC8208711 34134983

[3] Zhang H Chen K Tan Q Shao Q Han S Zhang C. Structural basis for chemokine recognition and receptor activation of chemokine receptor CCR5. Nat Commun (2021) 12(1):4151. doi: 10.1038/s41467-021-24438-5 34230484 PMC8260604

[4] Burg JS Ingram JR Venkatakrishnan AJ Jude KM Dukkipati A Feinberg EN. Structural biology. Structural basis for chemokine recognition and activation of a viral G protein-coupled receptor. Science (2015) 347(6226):1113–7. doi: 10.1126/science.aaa5026 PMC4445376 25745166

[5] Liu K Wu L Yuan S Wu M Xu Y Sun Q. Structural basis of CXC chemokine receptor 2 activation and signalling. Nature (2020) 585(7823):135–40. doi: 10.1038/s41586-020-2492-5 32610344

[6] Kofuku Y Yoshiura C Ueda T Terasawa H Hirai T Tominaga S. Structural basis of the interaction between chemokine stromal cell-derived factor-1/CXCL12 and its G-protein-coupled receptor CXCR4. J Biol Chem (2009) 284(50):35240–50. doi: 10.1074/jbc.M109.024851 PMC2787383 19837984

[7] Ravindran A Joseph PR Rajarathnam K. Structural basis for differential binding of the interleukin-8 monomer and dimer to the CXCR1 N-domain: role of coupled interactions and dynamics. Biochemistry (2009) 48(37):8795–805. doi: 10.1021/bi901194p PMC2858980 19681642

[8] Hughes CE Nibbs RJB. A guide to chemokines and their receptors. FEBS J (2018) 285(16):2944–71. doi: 10.1111/febs.14466 PMC6120486 29637711

[9] Yen YC Eberlie SA Dominik PK Deneka D Zhang P Schall TJ. Structures of atypical chemokine receptor 3 reveal the basis for its promiscuity and signaling bias. Sci Adv (2022) 8(28):eabn8063. doi: 10.1126/sciadv.abn8063 35857509 PMC9278869

[10] Smith JS Alagesan P Desai NK Pack TF Wu JH Inoue A. C-X-C motif chemokine receptor 3 splice variants differentially activate beta-arrestins to regulate downstream signaling pathways. Mol Pharmacol (2017) 92(2):136–50. doi: 10.1124/mol.117.108522 PMC5508197 28559424

[11] Gurevich VV Gurevich EV. Arrestins: critical players in trafficking of many GPCRs. Prog Mol Biol Transl Sci (2015) 132:1–14. doi: 10.1016/bs.pmbts.2015.02.010 26055052 PMC5841159

[12] Gurevich VV Gurevich EV. Arrestin-mediated signaling: Is there a controversy? World J Biol Chem (2018) 9(3):25–35. doi: 10.4331/wjbc.v9.i3.25 30595812 PMC6305498

[13] Bennett LD Fox JM Signoret N. Mechanisms regulating chemokine receptor activity. Immunology (2011) 134(3):246–56. doi: 10.1111/j.1365-2567.2011.03485.x PMC3209565 21977995

[14] Aramori I Ferguson SS Bieniasz PD Zhang J Cullen B Cullen MG. Molecular mechanism of desensitization of the chemokine receptor CCR-5: receptor signaling and internalization are dissociable from its role as an HIV-1 co-receptor. EMBO J (1997) 16(15):4606–16. doi: 10.1093/emboj/16.15.4606 PMC1170087 9303305

[15] Haribabu B Richardson RM Fisher I Sozzani S Peiper SC Horuk R. Regulation of human chemokine receptors CXCR4. Role of phosphorylation in desensitization and internalization. J Biol Chem (1997) 272(45):28726–31. doi: 10.1074/jbc.272.45.28726 9353342

[16] Grimm MC Ben-Baruch A Taub DD Howard OM Resau JH Wang JM. Opiates transdeactivate chemokine receptors: delta and mu opiate receptor-mediated heterologous desensitization. J Exp Med (1998) 188(2):317–25. doi: 10.1084/jem.188.2.317 PMC2212445 9670044

[17] Steele AD Szabo I Bednar F Rogers TJ. Interactions between opioid and chemokine receptors: heterologous desensitization. Cytokine Growth Factor Rev (2002) 13(3):209–22. doi: 10.1016/S1359-6101(02)00007-2 12486875

[18] Imai T Baba M Nishimura M Kakizaki M Takagi S Yoshie O. The T cell-directed CC chemokine TARC is a highly specific biological ligand for CC chemokine receptor 4. J Biol Chem (1997) 272(23):15036–42. doi: 10.1074/jbc.272.23.15036 9169480

[19] Imai T Chantry D Raport CJ Wood CL Nishimura M Godiska R. Macrophage-derived chemokine is a functional ligand for the CC chemokine receptor 4. J Biol Chem (1998) 273(3):1764–8. doi: 10.1074/jbc.273.3.1764 9430724

[20] Mariani M Lang R Binda E Panina-Bordignon P D'Ambrosio D. Dominance of CCL22 over CCL17 in induction of chemokine receptor CCR4 desensitization and internalization on human Th2 cells. Eur J Immunol (2004) 34(1):231–40. doi: 10.1002/eji.200324429 14971049

[21] Szabo I Chen XH Xin L Adler MW Howard OM Oppenheim JJ. Heterologous desensitization of opioid receptors by chemokines inhibits chemotaxis and enhances the perception of pain. Proc Natl Acad Sci U.S.A. (2002) 99(16):10276–81. doi: 10.1073/pnas.102327699 PMC124904 12130663

[22] Chen X Geller EB Rogers TJ Adler MW. Rapid heterologous desensitization of antinociceptive activity between mu or delta opioid receptors and chemokine receptors in rats. Drug Alcohol Depend (2007) 88(1):36–41. doi: 10.1016/j.drugalcdep.2006.09.010 17049756 PMC1880888

[23] Rajagopal S Bassoni DL Campbell JJ Gerard NP Gerard C Wehrman TS. Biased agonism as a mechanism for differential signaling by chemokine receptors. J Biol Chem (2013) 288(49):35039–48. doi: 10.1074/jbc.M113.479113 PMC3853256 24145037

[24] Eiger DS Boldizsar N Honeycutt CC Gardner J Rajagopal S. Biased agonism at chemokine receptors. Cell Signal (2021) 78:109862. doi: 10.1016/j.cellsig.2020.109862 33249087 PMC7775275

[25] Anderson CA Solari R Pease JE. Biased agonism at chemokine receptors: obstacles or opportunities for drug discovery? J Leukoc Biol (2016) 99(6):901–9. doi: 10.1189/jlb.2MR0815-392R 26701135

[26] Quoyer J Janz JM Luo J Ren Y Armando S Lukashova V. Pepducin targeting the C-X-C chemokine receptor type 4 acts as a biased agonist favoring activation of the inhibitory G protein. Proc Natl Acad Sci U.S.A. (2013) 110(52):E5088–97. doi: 10.1073/pnas.1312515110 PMC3876208 24309376

[27] Smith JS Nicholson LT Suwanpradid J Glenn RA Knape NM Alagesan P. Biased agonists of the chemokine receptor CXCR3 differentially control chemotaxis and inflammation. Sci Signal (2018) 11(555). doi: 10.1126/scisignal.aaq1075 PMC6329291 30401786

[28] Milanos L Brox R Frank T Poklukar G Palmisano R Waibel R. Discovery and characterization of biased allosteric agonists of the chemokine receptor CXCR3. J Med Chem (2016) 59(5):2222–43. doi: 10.1021/acs.jmedchem.5b01965 26862767

[29] Corbisier J Huszagh A Galés C Parmentier M Springael JY. Partial agonist and biased signaling properties of the synthetic enantiomers J113863/UCB35625 at chemokine receptors CCR2 and CCR5. J Biol Chem (2017) 292(2):575–84. doi: 10.1074/jbc.M116.757559 PMC5241733 27895119

[30] Sanchez J Lane JR Canals M Stone MJ. Influence of chemokine N-terminal modification on biased agonism at the chemokine receptor CCR1. Int J Mol Sci (2019) 20(10). doi: 10.3390/ijms20102417 PMC6566870 31096719

[31] Steen A Thiele S Guo D Hansen LS Frimurer TM Rosenkilde MM. Biased and constitutive signaling in the CC-chemokine receptor CCR5 by manipulating the interface between transmembrane helices 6 and 7. J Biol Chem (2013) 288(18):12511–21. doi: 10.1074/jbc.M112.449587 PMC3642299 23493400

[32] Shao Z Shen Q Yao B Mao C Chen LN Zhang H. Identification and mechanism of G protein-biased ligands for chemokine receptor CCR1. Nat Chem Biol (2022) 18(3):264–71. doi: 10.1038/s41589-021-00918-z PMC8885419 34949837

[33] Gao X Abdelkarim H Albee LJ Volkman BF Gaponenko V Majetschak M. Partial agonist activity of alpha1-adrenergic receptor antagonists for chemokine (C-X-C motif) receptor 4 and atypical chemokine receptor 3. PloS One (2018) 13(9):e0204041. doi: 10.1371/journal.pone.0204041 30248140 PMC6152952

[34] Meyrath M Reynders N Uchanski T Chevigne A Szpakowska M. Systematic reassessment of chemokine-receptor pairings confirms CCL20 but not CXCL13 and extends the spectrum of ACKR4 agonists to CCL22. J Leukoc Biol (2021) 109(2):373–6. doi: 10.1002/JLB.2AB0520-275R 32480426

[35] Martinelli R Sabroe I LaRosa G Williams TJ Pease JE. The CC chemokine eotaxin (CCL11) is a partial agonist of CC chemokine receptor 2b. J Biol Chem (2001) 276(46):42957–64. doi: 10.1074/jbc.M103933200 11559700

[36] Fox JM Najarro P Smith GL Struyf S Proost P Pease JE. Structure/function relationships of CCR8 agonists and antagonists. Amino-terminal extension of CCL1 by a single amino acid generates a partial agonist. J Biol Chem (2006) 281(48):36652–61. doi: 10.1074/jbc.M605584200 17023422

[37] Kiss DL Longden J Fechner GA Avery VM. The functional antagonist Met-RANTES: a modified agonist that induces differential CCR5 trafficking. Cell Mol Biol Lett (2009) 14(4):537–47. doi: 10.2478/s11658-009-0017-1 PMC6275935 19448977

[38] Capoulade-Metay C Ayouba A Kfutwah A Lole K Pêtres S Dudoit Y. A natural CCL5/RANTES variant antagonist for CCR1 and CCR3. Immunogenetics (2006) 58(7):533–41. doi: 10.1007/s00251-006-0133-2 16791620

[39] Harrison JK Fong AM Swain PA Chen S Yu YR Salafranca MN. Mutational analysis of the fractalkine chemokine domain. Basic amino acid residues differentially contribute to CX3CR1 binding, signaling, and cell adhesion. J Biol Chem (2001) 276(24):21632–41. doi: 10.1074/jbc.M010261200 11278650

[40] Nibbs RJ Salcedo TW Campbell JD Yao XT Li Y Nardelli B. C-C chemokine receptor 3 antagonism by the beta-chemokine macrophage inflammatory protein 4, a property strongly enhanced by an amino-terminal alanine-methionine swap. J Immunol (2000) 164(3):1488–97. doi: 10.4049/jimmunol.164.3.1488 10640766

[41] Getschman AE Imai Y Larsen O Peterson FC Wu X Rosenkilde MM. Protein engineering of the chemokine CCL20 prevents psoriasiform dermatitis in an IL-23-dependent murine model. Proc Natl Acad Sci U.S.A. (2017) 114(47):12460–5. doi: 10.1073/pnas.1704958114 PMC5703275 29109267

[42] Severin IC Souza AL Davis JH Musolino N Mack M Power CA. Properties of 7ND-CCL2 are modulated upon fusion to Fc. Protein Eng Des Sel (2012) 25(5):213–22. doi: 10.1093/protein/gzs008 22388887

[43] Veldkamp CT Seibert C Peterson FC De la Cruz NB Haugner JC 3rd Basnet H. Structural basis of CXCR4 sulfotyrosine recognition by the chemokine SDF-1/CXCL12. Sci Signal (2008) 1(37):ra4. doi: 10.1126/scisignal.1160755 18799424 PMC2692298

[44] Scholten DJ Canals M Wijtmans M de Munnik S Nguyen P Verzijl D. Pharmacological characterization of a small-molecule agonist for the chemokine receptor CXCR3. Br J Pharmacol (2012) 166(3):898–911. doi: 10.1111/j.1476-5381.2011.01648.x 21883151 PMC3417417

[45] Sachpatzidis A Benton BK Manfredi JP Wang H Hamilton A Dohlman HG. Identification of allosteric peptide agonists of CXCR4. J Biol Chem (2003) 278(2):896–907. doi: 10.1074/jbc.M204667200 12417595

[46] Garcia-Perez J Rueda P Staropoli I Kellenberger E Alcami J Arenzana-Seisdedos F. New insights into the mechanisms whereby low molecular weight CCR5 ligands inhibit HIV-1 infection. J Biol Chem (2011) 286(7):4978–90. doi: 10.1074/jbc.M110.168955 PMC3037610 21118814

[47] Qi B Fang Q Liu S Hou W Li J Huang Y. Advances of CCR5 antagonists: From small molecules to macromolecules. Eur J Med Chem (2020) 208:112819. doi: 10.1016/j.ejmech.2020.112819 32947226

[48] Crees ZD Rettig MP Bashey A Devine SM Jaglowski SM Wan F. Hematopoietic stem cell mobilization for allogeneic stem cell transplantation by motixafortide, a novel CXCR4 inhibitor. Blood Adv (2023) 7(12). doi: 10.1182/bloodadvances.2023010407 PMC10500469 37327120

[49] Sokkar P Harms M Sturzel C Gilg A Kizilsavas G Raasholm M. Computational modeling and experimental validation of the EPI-X4/CXCR4 complex allows rational design of small peptide antagonists. Commun Biol (2021) 4(1):1113. doi: 10.1038/s42003-021-02638-5 34552197 PMC8458281

[50] Mona CE Besserer-Offroy E Cabana J Lefrancois M Boulais PE Lefebvre MR. Structure-activity relationship and signaling of new chimeric CXCR4 agonists. J Med Chem (2016) 59(16):7512–24. doi: 10.1021/acs.jmedchem.6b00566 27434274

[51] Evans BJ Wang Z Broach JR Oishi S Fujii N Peiper SC. Expression of CXCR4, a G-protein-coupled receptor for CXCL12 in yeast identification of new-generation inverse agonists. Methods Enzymol (2009) 460:399–412. doi: 10.1016/S0076-6879(09)05220-3 19446737

[52] Wan Y Jakway JP Qiu H Shah H Garlisi CG Tian F. Identification of full, partial and inverse CC chemokine receptor 3 agonists using [35S]GTPgammaS binding. Eur J Pharmacol (2002) 456(1-3):1–10. doi: 10.1016/s0014-2999(02)02621-3 12450563

[53] Scholten DJ Wijtmans M van Senten JR Custers H Stunnenberg A de Esch IJ. Pharmacological characterization of [3H]VUF11211, a novel radiolabeled small-molecule inverse agonist for the chemokine receptor CXCR3. Mol Pharmacol (2015) 87(4):639–48. doi: 10.1124/mol.114.095265 25576486

[54] Rummel PC Arfelt KN Baumann L Jenkins TJ Thiele S Luttichau HR. Molecular requirements for inhibition of the chemokine receptor CCR8–probe-dependent allosteric interactions. Br J Pharmacol (2012) 167(6):1206–17. doi: 10.1111/j.1476-5381.2012.02076.x PMC3504988 22708643

[55] Bradley ME Bond ME Manini J Brown Z Charlton SJ. SB265610 is an allosteric, inverse agonist at the human CXCR2 receptor. Br J Pharmacol (2009) 158(1):328–38. doi: 10.1111/j.1476-5381.2009.00182.x PMC2795238 19422399

[56] Wasilko DJ Johnson ZL Ammirati M Che Y Griffor MC Han S. Structural basis for chemokine receptor CCR6 activation by the endogenous protein ligand CCL20. Nat Commun (2020) 11(1):3031. doi: 10.1038/s41467-020-16820-6 32541785 PMC7295996

[57] Tan Q Zhu Y Li J Chen Z Han GW Kufareva I. Structure of the CCR5 chemokine receptor-HIV entry inhibitor maraviroc complex. Science (2013) 341(6152):1387–90. doi: 10.1126/science.1241475 PMC3819204 24030490

[58] Urvas L Kellenberger E. Structural insights into molecular recognition and receptor activation in chemokine-chemokine receptor complexes. J Med Chem (2023) 66(11):7070–85. doi: 10.1021/acs.jmedchem.3c00352 37212620

[59] Gerard C Rollins BJ. Chemokines and disease. Nat Immunol (2001) 2(2):108–15. doi: 10.1038/84209 11175802

[60] Castan L Magnan A Bouchaud G. Chemokine receptors in allergic diseases. Allergy (2017) 72(5):682–90. doi: 10.1111/all.13089 27864967

[61] Pease JE. Targeting chemokine receptors in allergic disease. Biochem J (2011) 434(1):11–24. doi: 10.1042/BJ20101132 21269275

[62] Konrad FM Reutershan J. CXCR2 in acute lung injury. Mediators Inflamm 2012 (2012) 2012:740987. doi: 10.1155/2012/740987 PMC3375097 22719179

[63] Cui LY Chu SF Chen NH. The role of chemokines and chemokine receptors in multiple sclerosis. Int Immunopharmacol (2020) 83:106314. doi: 10.1016/j.intimp.2020.106314 32197226 PMC7156228

[64] Elemam NM Hannawi S Maghazachi AA. Role of chemokines and chemokine receptors in rheumatoid arthritis. Immunotargets Ther (2020) 9:43–56. doi: 10.2147/ITT.S243636 32211348 PMC7074856

[65] Proudfoot AE. Chemokine receptors: multifaceted therapeutic targets. Nat Rev Immunol (2002) 2(2):106–15. doi: 10.1038/nri722 PMC7097668 11910892

[66] Yang J Zheng Y Gou X Pu K Chen Z Guo Q. Prevalence of comorbidities and its effects in patients infected with SARS-CoV-2: a systematic review and meta-analysis. Int J Infect Dis (2020) 94:91–5. doi: 10.1016/j.ijid.2020.03.017 PMC7194638 32173574

[67] Noels H Weber C Koenen RR. Chemokines as therapeutic targets in cardiovascular disease. Arterioscler Thromb Vasc Biol (2019) 39(4):583–92. doi: 10.1161/ATVBAHA.118.312037 30760014

[68] Broder CC Collman RG. Chemokine receptors and HIV. J Leukoc Biol (1997) 62(1):20–9. doi: 10.1002/jlb.62.1.20 9225988

[69] Li G Fan Y Lai Y Han T Li Z Zhou P. Coronavirus infections and immune responses. J Med Virol (2020) 92(4):424–32. doi: 10.1002/jmv.25685 PMC7166547 31981224

[70] Bixler SL Goff AJ. The role of cytokines and chemokines in filovirus infection. Viruses (2015) 7(10):5489–507. doi: 10.3390/v7102892 PMC4632400 26512687

[71] Alcami A Lira SA. Modulation of chemokine activity by viruses. Curr Opin Immunol (2010) 22(4):482–7. doi: 10.1016/j.coi.2010.06.004 PMC6373461 20598516

[72] Domingo-Gonzalez R Prince O Cooper A Khader SA. Cytokines and chemokines in mycobacterium tuberculosis infection. Microbiol Spectr (2016) 4(5). doi: 10.1128/microbiolspec.TBTB2-0018-2016 PMC5205539 27763255

[73] Mollica Poeta V Massara M Capucetti A Bonecchi R. Chemokines and chemokine receptors: new targets for cancer immunotherapy. Front Immunol (2019) 10:379. doi: 10.3389/fimmu.2019.00379 30894861 PMC6414456

[74] Miao M De Clercq E Li G. Clinical significance of chemokine receptor antagonists. Expert Opin Drug Metab Toxicol (2020) 16(1):11–30. doi: 10.1080/17425255.2020.1711884 31903790

[75] Hitchinson B Eby JM Gao X Guite-Vinet F Ziarek JJ Abdelkarim H. Biased antagonism of CXCR4 avoids antagonist tolerance. Sci Signal (2018) 11(552). doi: 10.1126/scisignal.aat2214 PMC6422681 30327409

[76] Grozdanovic M Laffey KG Abdelkarim H Hitchinson B Harijith A Moon HG. Novel peptide nanoparticle-biased antagonist of CCR3 blocks eosinophil recruitment and airway hyperresponsiveness. J Allergy Clin Immunol (2019) 143(2):669–680 e12. doi: 10.1016/j.jaci.2018.05.003 29778505 PMC6240402

[77] Vincent J Dachman W Blaschke TF Hoffman BB. Pharmacological tolerance to alpha 1-adrenergic receptor antagonism mediated by terazosin in humans. J Clin Invest (1992) 90(5):1763–8. doi: 10.1172/JCI116050 PMC443234 1358918

[78] Komazawa Y Adachi K Mihara T Ono M Kawamura A Fujishiro H. Tolerance to famotidine and ranitidine treatment after 14 days of administration in healthy subjects without Helicobacter pylori infection. J Gastroenterol Hepatol (2003) 18(6):678–82. doi: 10.1046/j.1440-1746.2003.03041.x 12753150

[79] Olsen CK Brennum LT Kreilgaard M. Using pharmacokinetic-pharmacodynamic modelling as a tool for prediction of therapeutic effective plasma levels of antipsychotics. Eur J Pharmacol (2008) 584(2-3):318–27. doi: 10.1016/j.ejphar.2008.02.005 18325493

[80] Boyle AE Stewart RB Macenski MJ Spiga R Johnson BA Meisch RA. Effects of acute and chronic doses of naltrexone on ethanol self-administration in rhesus monkeys. Alcohol Clin Exp Res (1998) 22(2):359–66. doi: 10.1111/j.1530-0277.1998.tb03661.x 9581641

[81] LiverTox: Clinical and Research Information on Drug-Induced Liver Injury. Bethesda MD: Book published by NIH: National Institute for Digestive and Kidney diseases (2012).

[82] Jorgensen AS Daugvilaite V De Filippo K Berg C Mavri M Benned-Jensen T. Biased action of the CXCR4-targeting drug plerixafor is essential for its superior hematopoietic stem cell mobilization. Commun Biol (2021) 4(1):569. doi: 10.1038/s42003-021-02070-9 33980979 PMC8115334

[83] Lagresle-Peyrou C Lefrère F Magrin E Ribeil JA Romano O Weber L. Plerixafor enables safe, rapid, efficient mobilization of hematopoietic stem cells in sickle cell disease patients after exchange transfusion. Haematologica (2018) 103(5):778–86. doi: 10.3324/haematol.2017.184788 PMC5927997 29472357

[84] Yoshie O. CCR4 as a therapeutic target for cancer immunotherapy. Cancers (Basel) (2021) 13(21). doi: 10.3390/cancers13215542 PMC8583476 34771703

[85] Duvic M Evans M Wang C. Mogamulizumab for the treatment of cutaneous T-cell lymphoma: recent advances and clinical potential. Ther Adv Hematol (2016) 7(3):171–4. doi: 10.1177/2040620716636541 PMC4872175 27247757

[86] Ray N. Maraviroc in the treatment of HIV infection. Drug Des Devel Ther (2009) 2:151–61. doi: 10.2147/dddt.s3474 PMC2761192 19920903

[87] Swenson LC Mo T Dong WW Zhong X Woods CK Jensen MA. Deep sequencing to infer HIV-1 co-receptor usage: application to three clinical trials of maraviroc in treatment-experienced patients. J Infect Dis (2011) 203(2):237–45. doi: 10.1093/infdis/jiq030 PMC3071057 21288824

[88] De Luca A Pezzotti P Boucher C Döring M Incardona F Kaiser R. Clinical use, efficacy, and durability of maraviroc for antiretroviral therapy in routine care: A European survey. PloS One (2019) 14(11):e0225381. doi: 10.1371/journal.pone.0225381 31751385 PMC6874206

[89] O’Byrne PM Metev H Puu M Richter K Keen C Uddin M. Efficacy and safety of a CXCR2 antagonist, AZD5069, in patients with uncontrolled persistent asthma: a randomised, double-blind, placebo-controlled trial. Lancet Respir Med (2016) 4(10):797–806. doi: 10.1016/S2213-2600(16)30227-2 27574788

[90] Gale JD Berger B Gilbert S Popa S Sultan MB Schachar RA. A CCR2/5 inhibitor, PF-04634817, is inferior to monthly ranibizumab in the treatment of diabetic macular edema. Invest Ophthalmol Vis Sci (2018) 59(6):2659–69. doi: 10.1167/iovs.17-22731 29847672

[91] Ushijima I Mizuki Y Yamada M. Development of tolerance and reverse tolerance to haloperidol- and SCH23390-induced cataleptic effects during withdrawal periods after long-term treatment. Pharmacol Biochem Behav (1995) 50(2):259–64. doi: 10.1016/0091-3057(94)00309-7 7740066

[92] Kitaichi K Yamada K Yoneda Y Ogita K Hasegawa T Furukawa H. Risperidone prevents the development of supersensitivity, but not tolerance, to phencyclidine in rats treated with subacute phencyclidine. Life Sci (1995) 56(7):531–43. doi: 10.1016/0024-3205(94)00482-8 7532775

[93] Ozcan S Soydan A Tamam L. Supersensitivity psychosis in a case with clozapine tolerance. Eur Rev Med Pharmacol Sci (2012) 16 Suppl 4:70–3. 23090814

[94] Hancock AA. The challenge of drug discovery of a GPCR target: analysis of preclinical pharmacology of histamine H3 antagonists/inverse agonists. Biochem Pharmacol (2006) 71(8):1103–13. doi: 10.1016/j.bcp.2005.10.033 16513092

[95] Smit MJ Leurs R Alewijnse AE Blauw J Amerongen Nieuw Van GP Vrede De Van Y. Inverse agonism of histamine H2 antagonist accounts for upregulation of spontaneously active histamine H2 receptors. Proc Natl Acad Sci U.S.A. (1996) 93(13):6802–7. doi: 10.1073/pnas.93.13.6802 PMC39108 8692899

[96] Khilnani G Khilnani AK. Inverse agonism and its therapeutic significance. Indian J Pharmacol (2011) 43(5):492–501. doi: 10.4103/0253-7613.84947 22021988 PMC3195115

[97] Nishimura Y Ii M Qin G Hamada H Asai J Takenaka H. CXCR4 antagonist AMD3100 accelerates impaired wound healing in diabetic mice. J Invest Dermatol (2012) 132(3 Pt 1):711–20. doi: 10.1038/jid.2011.356 PMC3276738 22048734

[98] Xu H Zhu Z Huang Y Ildstad ST. FL/GCSF/AMD3100-mobilized hematopoietic stem cells induce mixed chimerism with nonmyeloablative conditioning and transplantation tolerance. Transplantation (2019) 103(7):1360–71. doi: 10.1097/TP.0000000000002657 30747856

[99] Woollard SM Kanmogne GD. Maraviroc: a review of its use in HIV infection and beyond. Drug Des Devel Ther (2015) 9:5447–68. doi: 10.2147/DDDT.S90580 PMC4598208 26491256

[100] Schall TJ Proudfoot AE. Overcoming hurdles in developing successful drugs targeting chemokine receptors. Nat Rev Immunol (2011) 11(5):355–63. doi: 10.1038/nri2972 21494268

[101] Horuk R. Chemokine receptor antagonists: overcoming developmental hurdles. Nat Rev Drug Discovery (2009) 8(1):23–33. doi: 10.1038/nrd2734 19079127

[102] Raehal KM Schmid CL Groer CE Bohn LM. Functional selectivity at the mu-opioid receptor: implications for understanding opioid analgesia and tolerance. Pharmacol Rev (2011) 63(4):1001–19. doi: 10.1124/pr.111.004598 PMC3186080 21873412

[103] Deng HB Yu Y Pak Y O'Dowd BF George SR Surratt CK. Role for the C-terminus in agonist-induced mu opioid receptor phosphorylation and desensitization. Biochemistry (2000) 39(18):5492–9. doi: 10.1021/bi991938b 10820022

[104] Salin-Pascual RJ Granados-Fuentes D Galicia-Polo L Nieves E Gillin JC. Development of tolerance after repeated administration of a selective muscarinic M1 antagonist biperiden in healthy human volunteers. Biol Psychiatry (1993) 33(3):188–93. doi: 10.1016/0006-3223(93)90138-4 8448266

[105] Villanueva HF Porter JH. Differential tolerance to the behavioral effects of chronic pimozide and clozapine on multiple random interval responding in rats. Behav Pharmacol (1993) 4(3):201–8. doi: 10.1097/00008877-199306000-00002 11224187

[106] Varvel SA Vann RE Wise LE Philibin SD Porter JH. Effects of antipsychotic drugs on operant responding after acute and repeated administration. Psychopharmacol (Berl) (2002) 160(2):182–91. doi: 10.1007/s00213-001-0969-y 11875636

[107] Claus A Bollen J Cuyper De H Eneman M Malfroid M Peuskens J. Risperidone versus haloperidol in the treatment of chronic schizophrenic inpatients: a multicentre double-blind comparative study. Acta Psychiatr Scand (1992) 85(4):295–305. doi: 10.1111/j.1600-0447.1992.tb01473.x 1375801

[108] Gao J Li M. Differential effects of intermittent versus continuous haloperidol treatment throughout adolescence on haloperidol sensitization and social behavior in adulthood. Prog Neuropsychopharmacol Biol Psychiatry (2014) 54:67–75. doi: 10.1016/j.pnpbp.2014.05.015 24942467 PMC4134967

[109] Yen YC Lung FW Chong MY. Adverse effects of risperidone and haloperidol treatment in schizophrenia. Prog Neuropsychopharmacol Biol Psychiatry (2004) 28(2):285–90. doi: 10.1016/j.pnpbp.2003.10.006 14751424

[110] Nyberg S Farde L Eriksson L Halldin C Eriksson B. 5-HT2 and D2 dopamine receptor occupancy in the living human brain. A PET study with risperidone. Psychopharmacol (Berl) (1993) 110(3):265–72. doi: 10.1007/BF02251280 7530376

[111] Wanka L Behr V Beck-Sickinger AG. Arrestin-dependent internalization of rhodopsin-like G protein-coupled receptors. Biol Chem (2022) 403(2):133–49. doi: 10.1515/hsz-2021-0128 34036761

[112] Sorkin A Von Zastrow M. Signal transduction and endocytosis: close encounters of many kinds. Nat Rev Mol Cell Biol (2002) 3(8):600–14. doi: 10.1038/nrm883 12154371

[113] Kleinau G Muller A Biebermann H. Oligomerization of GPCRs involved in endocrine regulation. J Mol Endocrinol (2016) 57(1):R59–80. doi: 10.1530/JME-16-0049 27151573

[114] Thelen M Munoz LM Rodriguez-Frade JM Mellado M. Chemokine receptor oligomerization: functional considerations. Curr Opin Pharmacol (2010) 10(1):38–43. doi: 10.1016/j.coph.2009.09.004 19828377

[115] O’Dowd BF Alijaniaram M Ji X Nguyen T Eglen RM George SR. Using ligand-induced conformational change to screen for compounds targeting G-protein-coupled receptors. J Biomol Screen (2007) 12(2):175–85. doi: 10.1177/1087057106298287 17289935

[116] Hess EJ Norman AB Creese I. Chronic treatment with dopamine receptor antagonists: behavioral and pharmacologic effects on D1 and D2 dopamine receptors. J Neurosci (1988) 8(7):2361–70. doi: 10.1523/JNEUROSCI.08-07-02361.1988 PMC6569513 2907912

[117] Milligan G Ward RJ Marsango S. GPCR homo-oligomerization. Curr Opin Cell Biol (2019) 57:40–7. doi: 10.1016/j.ceb.2018.10.007 PMC7083226 30453145

[118] Sleno R Hebert TE. The dynamics of GPCR oligomerization and their functional consequences. Int Rev Cell Mol Biol (2018) 338:141–71. doi: 10.1016/bs.ircmb.2018.02.005 29699691

[119] Munoz LM Holgado BL Martinez AC Rodriguez-Frade JM Mellado M. Chemokine receptor oligomerization: a further step toward chemokine function. Immunol Lett (2012) 145(1-2):23–9. doi: 10.1016/j.imlet.2012.04.012 22698180

[120] Panetta R Greenwood MT. Physiological relevance of GPCR oligomerization and its impact on drug discovery. Drug Discovery Today (2008) 13(23-24):1059–66. doi: 10.1016/j.drudis.2008.09.002 18824244

[121] Casado V Cortes A Mallol J Perez-Capote K Ferre S Lluis C. GPCR homomers and heteromers: a better choice as targets for drug development than GPCR monomers? Pharmacol Ther (2009) 124(2):248–57. doi: 10.1016/j.pharmthera.2009.07.005 PMC9386294 19664655

[122] Qin L Kufareva I Holden LG Wang C Zheng Y Zhao C. Structural biology. Crystal structure of the chemokine receptor CXCR4 in complex with a viral chemokine. Science (2015) 347(6226):1117–22. doi: 10.1126/science.1261064 PMC4362693 25612609

[123] Fuxe K Ferre S Genedani S Franco R Agnati LF. Adenosine receptor-dopamine receptor interactions in the basal ganglia and their relevance for brain function. Physiol Behav (2007) 92(1-2):210–7. doi: 10.1016/j.physbeh.2007.05.034 17572452

[124] Ferre S Quiroz C Woods AS Cunha R Popoli P Ciruela F. An update on adenosine A2A-dopamine D2 receptor interactions: implications for the function of G protein-coupled receptors. Curr Pharm Des (2008) 14(15):1468–74. doi: 10.2174/138161208784480108 PMC2424285 18537670

[125] Bonaventura J Navarro G Casado-Anguera V Azdad K Rea W Moreno E. Allosteric interactions between agonists and antagonists within the adenosine A2A receptor-dopamine D2 receptor heterotetramer. Proc Natl Acad Sci U.S.A. (2015) 112(27):E3609–18. doi: 10.1073/pnas.1507704112 PMC4500251 26100888

[126] McHeik S Van Eeckhout N De Poorter C Gales C Parmentier M Springael JY. Coexpression of CCR7 and CXCR4 during B cell development controls CXCR4 responsiveness and bone marrow homing. Front Immunol (2019) 10:2970. doi: 10.3389/fimmu.2019.02970 31921208 PMC6930800

[127] Heuninck J Viciano Perpina C Isbilir A Caspar B Capoferri D Briddon SJ. Context-dependent signaling of CXC chemokine receptor 4 and atypical chemokine receptor 3. Mol Pharmacol (2019) 96(6):778–93. doi: 10.1124/mol.118.115477 31092552

[128] Watts AO van Lipzig MM Jaeger WC Seeber RM van Zwam M Vinet J. Identification and profiling of CXCR3-CXCR4 chemokine receptor heteromer complexes. Br J Pharmacol (2013) 168(7):1662–74. doi: 10.1111/bph.12064 PMC3605874 23170857

[129] Matthys P Hatse S Vermeire K Wuyts A Bridger G Henson GW. AMD3100, a potent and specific antagonist of the stromal cell-derived factor-1 chemokine receptor CXCR4, inhibits autoimmune joint inflammation in IFN-gamma receptor-deficient mice. J Immunol (2001) 167(8):4686–92. doi: 10.4049/jimmunol.167.8.4686 11591799

[130] Hatse S Princen K Bridger G De Clercq E Schols D. Chemokine receptor inhibition by AMD3100 is strictly confined to CXCR4. FEBS Lett (2002) 527(1-3):255–62. doi: 10.1016/S0014-5793(02)03143-5 12220670

[131] Kalatskaya I Berchiche YA Gravel S Limberg BJ Rosenbaum JS Heveker N. AMD3100 is a CXCR7 ligand with allosteric agonist properties. Mol Pharmacol (2009) 75(5):1240–7. doi: 10.1124/mol.108.053389 19255243

[132] Del Molino Del Barrio I Wilkins GC Meeson A Ali S Kirby JA. Breast cancer: an examination of the potential of ACKR3 to modify the response of CXCR4 to CXCL12. Int J Mol Sci (2018) 19(11). doi: 10.3390/ijms19113592 PMC6274818 30441765

[133] Murphy PM Heusinkveld L. Multisystem multitasking by CXCL12 and its receptors CXCR4 and ACKR3. Cytokine (2018) 109:2–10. doi: 10.1016/j.cyto.2017.12.022 29398278 PMC6003845

[134] Uto-Konomi A McKibben B Wirtz J Sato Y Takano A Nanki T. CXCR7 agonists inhibit the function of CXCL12 by down-regulation of CXCR4. Biochem Biophys Res Commun (2013) 431(4):772–6. doi: 10.1016/j.bbrc.2013.01.032 23333329

[135] Inaguma S Riku M Ito H Tsunoda T Ikeda H Kasai K. GLI1 orchestrates CXCR4/CXCR7 signaling to enhance migration and metastasis of breast cancer cells. Oncotarget (2015) 6(32):33648–57. doi: 10.18632/oncotarget.5203 PMC4741792 26413813

[136] Jastrzebska B Chen Y Orban T Jin H Hofmann L Palczewski K. Disruption of rhodopsin dimerization with synthetic peptides targeting an interaction interface. J Biol Chem (2015) 290(42):25728–44. doi: 10.1074/jbc.M115.662684 PMC4646215 26330551

[137] Botta J Bibic L Killoran P McCormick PJ Howell LA. Design and development of stapled transmembrane peptides that disrupt the activity of G-protein-coupled receptor oligomers. J Biol Chem (2019) 294(45):16587–603. doi: 10.1074/jbc.RA119.009160 PMC6851324 31467080

[138] Gallo M Defaus S Andreu D. Disrupting GPCR complexes with smart drug-like peptides. Pharmaceutics (2022) 14(1). doi: 10.3390/pharmaceutics14010161 PMC8779866 35057055

[139] Gallo M Navarro G Franco R Andreu D. A(2A) receptor homodimer-disrupting sequence efficiently delivered by a protease-resistant, cyclic CPP vector. Int J Mol Sci (2019) 20(19). doi: 10.3390/ijms20194937 PMC6801510 31590403

